# Unsupervised binning of environmental genomic fragments based on an error robust selection of *l*-mers

**DOI:** 10.1186/1471-2105-11-S2-S5

**Published:** 2010-04-16

**Authors:** Bin Yang, Yu Peng, Henry Chi-Ming Leung, Siu-Ming Yiu, Jing-Chi Chen, Francis Yuk-Lun Chin

**Affiliations:** 1 State Key Laboratory of Bioelectronics, School of Biological Science & Medical Engineering, Southeast University, Nanjing, Jiangsu, 210096 PR China; 2 Department of Computer Science, The University of Hong Kong, Pok Fu Lam Road, Hong Kong

## Abstract

**Background:**

With the rapid development of genome sequencing techniques, traditional research methods based on the isolation and cultivation of microorganisms are being gradually replaced by metagenomics, which is also known as environmental genomics. The first step, which is still a major bottleneck, of metagenomics is the taxonomic characterization of DNA fragments (reads) resulting from sequencing a sample of mixed species. This step is usually referred as “binning”. Existing binning methods are based on supervised or semi-supervised approaches which rely heavily on reference genomes of known microorganisms and phylogenetic marker genes. Due to the limited availability of reference genomes and the bias and instability of marker genes, existing binning methods may not be applicable in many cases.

**Results:**

In this paper, we present an unsupervised binning method based on the distribution of a carefully selected set of *l*-mers (substrings of length *l* in DNA fragments). From our experiments, we show that our method can accurately bin DNA fragments with various lengths and relative species abundance ratios without using any reference and training datasets.

Another feature of our method is its error robustness. The binning accuracy decreases by less than 1% when the sequencing error rate increases from 0% to 5%. Note that the typical sequencing error rate of existing commercial sequencing platforms is less than 2%.

**Conclusions:**

We provide a new and effective tool to solve the metagenome binning problem without using any reference datasets or markers information of any known reference genomes (species). The source code of our software tool, the reference genomes of the species for generating the test datasets and the corresponding test datasets are available at http://i.cs.hku.hk/~alse/MetaCluster/.

## Background

Microbes are essential for almost every process in the biosphere and for every part of human life in both the positive and negative sense, for example, the production of yoghurt with *Lactobacillus* and alcohol brewing with *yeast* as well as the fatal pathogen of pulmonary tuberculosis and cholera. The impact of microbes on humans is not limited to several kinds of species. The complex environment of human life is maintained by microbial communities which are composed of dozens to thousands kinds of individual microbes. The unbalance or abnormal diversity of these microbial communities is proved to be associated with common diseases like pericementitis [1] and gastrointestinal [2] disturbance. Understanding how microbial community diversity affects health and disease may contribute to better diagnosis, prevention, and treatment of diseases. During the last centuries, researches on microbes have been based on the isolation, cultivation and purification of individual microorganism from complex communities. But 99% of all microbial diversity in the biosphere, as yet, is uncultivable [3], because of the highly artificial and limited number of conditions used currently for cultivation. For the uncultivable majority of the microbial communities, rapidly developing genome sequencing techniques can help. *Metagenomics*, which is also known as the *environmental genomics*, applies the shotgun sequencing technique to mixed genome samples, obtained directly from an environmental sample or series of related samples, producing high-throughput randomly sampled DNA fragments of these genome samples [4]. Since 2004, several metagenomics sequencing projects have been successfully implemented, such as Acid Mine Drainage Biofilm (AMD) for dozens of species [5] and the recent Human Gut Microbiome (HGM) for more than thousands of species [6].

Different from traditional single genome sequencing researches where all DNA fragments are coming from one single species, the metagenomics sequencing dataset contains DNA fragments from different species where most of their genomes are unknown. The data analysis process of metagenomic sequencing (MS) dataset requires an additional analyzing step, called “binning” [7]. The binning step assigns the DNA fragments to the taxonomy tree which provides a general map of the microbe distribution of the mixed sample, basically answering the essential question of metagenomic research: what’s in the mix? Various phylogenetic resolution or taxonomical rank of binning from high level such as kingdom to low level such as genus depends on the research requirements and the quality of the MS dataset.

A number of currently available binning methods fall into two broad categories: sequence similarity-based and sequence composition-based. The first, for example based on BLAST [8], classifies fragments based on the distribution of BLAST hits of phylogenetic specific marker genes to taxonomic classes [9]. The application of this kind of method is limited due to the limited availability of reference genomes of known microorganisms. As mentioned above, less than 1% of all microorganisms can be cultured and sequenced today.

So more generic features such as structure and composition form the basis of methods developed to distinguish components from mixed sequencing dataset in a supervised or semi-supervised manner. In general, these methods extract the composition features of reference genomes or marker regions (e.g. the widely applied fingerprint gene *16S rRNA *[10], *recA* and *rpoB*). Then, a classifier is generated based on different machine learning methods like SVM or SOM with the selected training dataset. For semi-supervised methods, the marker information of the testing datasets is translated to additional constraints during the clustering or classification process. Compared to the sequence similarity-based methods, sequence composition-based methods achieve better performance. But there are still limitations on these approaches; For example, not all species inside the sample carry the known phylogenetic markers. According to several metagenomic projects, such as the enhanced biological phosphorus removing (EBPR) sludge [11], Sargasso Sea [4] and the Minnesota soil samples [12], only 0.17%, 0.06% and 0.017% of the contigs (fragments) respectively are known to carry *16S rRNA* markers. Even if we consider other markers such as *recA* and *rpoB*, less than 1% of the fragments could be identified. Also, some species may share multiple markers with other species, which leads to incorrect classifications. For example, according to recent reports, multiple kinds of *16S rRNA* molecules can exist in a single bacterium [13]. Moreover, the marker gene information is provided by the existing cultivation and isolation techniques with limitation and bias in the selection of specific microorganisms. The training datasets with the bias caused by technical limitation will also introduce bias into the classification and clustering results.

**Figure 1:**
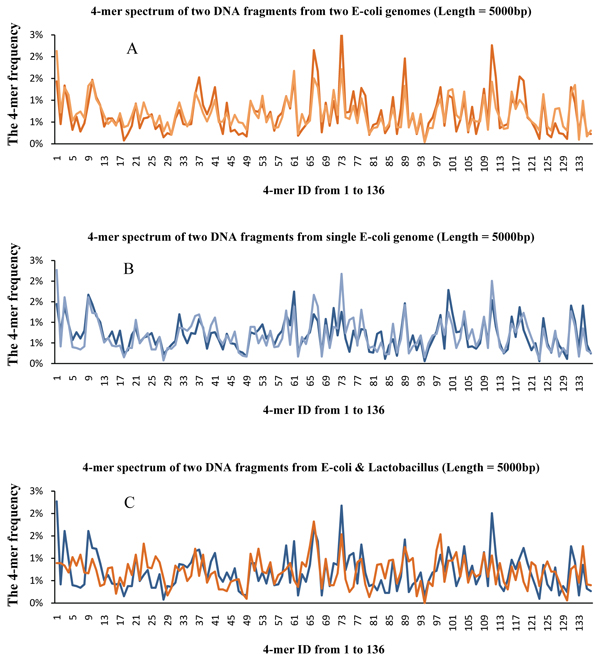
**The 4-mer frequency spectrum** (Figure. 1A) gives the 4-mer spectrums of two DNA fragments from two different E-coli genomes. (Figure. 1B) gives the 4-mer spectrums of two DNA fragments from single E-coli genome. (Figure. 1C) gives the 4-mer spectrums of two DNA fragments from the genomes of E-coli and Lactobacillus which belong to the same Kingdom but different Phylums.

These two categories of methods suffer from the limitation or instability of different kinds of reference information and not much research has been done for the clustering DNA fragments from species without genomes information [14]. To address this issue, we propose an unsupervised method for clustering DNA fragments based on *l*-mer (short DNA substrings of length *l* in the fragments) distribution [15-19]. Previous research of this compositional signature has shown that species belonging to different categories, even down to genus, tend to be quite distinct in terms of the *l*-mer frequency of their whole genomes or genome fragments [15,20]. So by comparing the *l*-mer distribution of fragments, we may be able to bin the DNA fragments into the correct taxonomical groups. Figure 1(A) shows the *l*-mer distributions of two species in the same genus while Figure 1(C) shows the *l*-mer distributions of two species in different genuses. The distributions are similar in 1(A) and are quite different in 1(C).

However, the difficulty of applying *l*-mer distribution lies in the resulting high-dimensional data. The *l*-mer frequency of each input DNA fragment is usually represented by a feature vector with 4*^l^* components. Consider the palindrome reverse and complement DNA string in the sequencing datasets, the dimension of the *l*-mer vector could be decreased to about 4*^l^*/2. Details of the generation of the *l*-mer vectors will be described late in the Methods Section. When *l* ≥ 4, the dimension of the feature vector is very large (i.e. when *l* = 4, the dimension of the feature vector is 136) and the clustering problem based on the high dimensional feature vectors becomes difficult. Some researchers suggested using methods like PCA (Principal Components Analysis) etc. to come up with a combination of selected significant *l*-mers [21] to lower the dimension. However, we show that some seemingly significant *l*-mers may be due to noise, and simply applying PCA cannot filter out this noise. Moreover, PCA involves a complicated process and it is not easy to understand the resulting combination of *l*-mers (in terms of eigenvectors) and to find the biological meaning of the eigenvectors. Thus, selecting an appropriate set of *l*-mers to decrease the dimension of the datasets poses a difficult problem.

To tackle this problem, we introduce a simple but error-robust method, based on a modified Chebychev Distance, to decrease the dimension of the dataset by remove some *l*-mers. Our selection of *l*-mers combined with a simple *k*-mean clustering algorithm is shown to be effective in the binning process.

Note that our method does not rely on any reference sequence or training dataset, but is only based on the similarity of the *l*-mer frequencies. Our method could bin the raw DNA fragments to several taxonomy specific clusters with high accuracy and resolution. The other advantage of our method is its robustness with respect to sequencing errors. The binning performance decreases by less than 1% while the sequencing error rate increases from 0% to 5% which is much higher than the typical sequencing error rate of less than 2% for the existing commercial sequencing platforms. We believe that our approach is promising to solve the metagenomics binning problem for short fragments generated by the high-throughput sequencing machines.

## Methods

**Figure 2:**
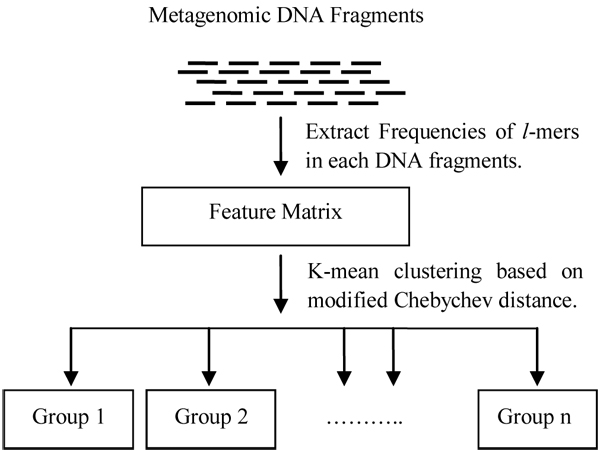
**The pipeline of our binning algorithm** First, the *l*-mer frequencies of each input sequence are counted. Then based on a novel modified Chebychev distance, a subset of *l*-mers is selected to create a feature vector. After that, *k*-mean clustering algorithm is applied to classify the fragments into taxonomic specific groups.

The approach of our binning method is outlined in Figure 2. First, the *l*-mer occurrence frequencies of each DNA fragment in the sample are counted. Not all *l*-mers will be used in the classification process. We have a simple, but effective method, based on our novel modified Chebychev distance, to select a subset of *l*-mers for a feature vector, which is 20% of the raw dimension. After that, a *k*-mean algorithm is applied to classify the fragments into different taxonomical groups.

### *l*-mer frequency calculation

The *DNA composition features* of each DNA fragment are extracted by calculating its *l*-mer frequencies. There are 4 different nucleotides in a DNA sequence, so there are in total 4*^l^l*-mers. A scan window of length *l* is slid along each DNA fragment and the frequency of every *l*-mer, say Ni, i ∈ [1, 4*^l^*], is recorded. For practice, DNA fragments are of different lengths and they contain different numbers of *l*-mers, for example, a DNA fragment of length 500bp contains 497 4-mers and a DNA fragment of length 2000bp contains 1997 4-mers. So we cannot compare directly the *l*-mer frequencies of two DNA fragments of different lengths. We need to apply an extra step to normalize the *l*-mer frequencies based on the lengths of the DNA fragments. Set the total number of *l*-mers in a DNA fragment be: , the normalized frequency of each *l*-mer is . Then the *feature vector* is defined as  with 4*^l^* components. After getting the *l*-mer frequencies, we need to do some modification to make them applicable for reverse complement strings. As each DNA fragment can be sequenced from either strand of the DNA genome, they should give the same *l*-mer frequencies. Hence, we can combine the frequency of one *l*-mer and its reverse complement palindrome *l*-mer into a single frequency for counting. This process will reduce the size of vector by half, i.e. the size of the vector for *l*-mer , if *l* is odd; , if *l* is even.

Based on some previous studies [22,23], in order to be effective and to have a reasonable vector size, *l* is set to 4. So each DNA fragment will be transformed to a vector with 136 components and the input sequencing dataset of FASTA will be transformed to a *n* × 136  matrix with *n* rows representing *n* DNA fragments.

### Modified Chebychev Distance

Clustering high-dimensional vectors (representing the DNA fragments) of 136 values is not easy. Because of noise, not all the *l*-mers (components in the feature vectors) are useful. Based on our experiments and analysis, two kinds of major noises *l*-mers are identified, the *intraspecies noise l*-mers (about 20% of the total *l*-mers) and *interspecies noise l*-mers (about 60% of the total *l*-mers). In the following parts of this section, we will introduce how to identify and remove these two kinds of noises.

Even though the high-throughput sequencing technology and the assembly process could provide DNA fragments covering the whole genome, because of the noise in data, even for the same genome of the same species, the *l*-mer distribution of a general region of the genome may be quite different from the *l*-mer distribution of a special functional region (such as promoters and exogenous transferred regions). This is called *intraspecies noise*. Figure 1(B) shows an example of two regions from the same genome. These two regions have quite different *l*-mer distributions (compared to Figure 1(A)). However, this does not occur very often. Usually randomly picked regions from the same genome should have quite similar *l*-mer distributions, as shown in Figure 1(A). The following procedure can be used to remove these outliners.

We first define a *Modified Chebychev Distance* which combines the idea of Chebychev and Manhattan distance to represent the similarity between a pair of feature vectors. Let *a* and *b* be two feature vectors where *a_i _* and *b_i _* are their *i ^th^* components. The traditional Chebychev Distance calculates the maximum absolute difference of each component in these two vectors, i.e.

Chebychev Distance examines the differences across all components of the vectors and uses only the maximum difference as the measure. However the extreme values are most likely caused by intraspecies noise. To avoid using these noisy *l*-mers as measure, we construct the *sorted value list* of *a* and *b* where the values of  are sorted in increasing order, i.e. if 4-mers are taken as an example,  is the minimum and  is the maximum. Then the last 20% of the top values are removed as intraspecies noise. The 20% cut off value is determined based on extensive experiments and how to determine this value should be further investigated, but in general, the results are similar for choosing the cut off values between 15% and 25%.

The other type of noise called *interspecies noise* is due to similar and statistical unstable *l*-mers among interspecies. Within the entire 136 4-mers, only a few of them are essential and effective for representing the distance (similarity) between the feature vectors of two DNA fragments. Hence, any distance definitions which consider all the *l*-mers will introduce noises caused by these “unessential” *l*-mers, which lead to unsatisfactory results.

**Figure 3:**
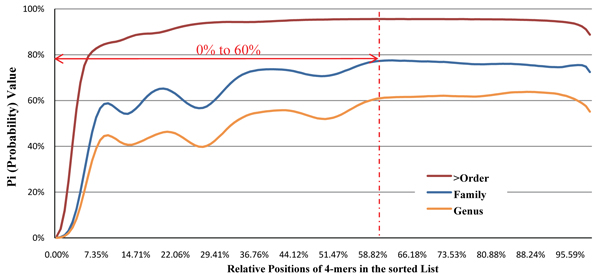
**The filter out range of unessential 4-mer selection** Not all the *l*-mers are essential for defining the similarity between two DNA fragments. In figure 3, the x-axis represents the relative position of the *D_i _* values in the sorted value list and the y-axis represents the value of *P_i _*, which could be considered as the confidence level or the validity of the selection of corresponding *D_i _* value as distance for species discrimination. We filter out the range from 0% to 60% to achieve better performance.

A group of experiments were conducted to identify those unessential *l*-mers to be removed for calculating the distance between the feature vectors of two DNA fragments. Let *D_i _* be its corresponding  value. Without loss of generality, assume that *D*_1_ is the minimum value and *D*_136_ the maximum (if 4-mers are taken as an example). The experiments were conducted based on the reference genomes of some known species or microorganisms in NCBI genome database. Three DNA fragments, say ,  and , were picked randomly where  and  are from the same species and  from a different species within a particular taxonomical differentia level. Let , the *i ^th^* element of the sorted value list of *X* and *Y*. The interspecies distance *D_i _*(*A*, *C*)

and the intraspecies distance *D_i _*(*A*, *B*) were then compared. This process was repeated many times with different sets of three DNA fragments. Basically we want to determine whether the *i ^th^l*-mer is essential in calculating distance between fragments from same/different species. *P_i _* (Probability) is used to indicate the ratio of the total number of experiments that interspecies distance *D_i _*(*A*, *C*) is larger than the intraspecies distance *D_i _*(*A*, *B*). Figure 3 give the corresponding *P_i _* for each *D_i _* in the sorted value list of 1 million experiments on data from NCBI genomes database.

Three sets of experiments were conducted to generate the three curves in Figure 3. Each curve represents a particular taxonomical differentia level among DNA fragment  and . We tested the differentia level at “Genus” ( and  are from the same family but different genuses), “Family” ( and  are from the same order but different families) and “> Order” ( and  are from same class or higher taxonomical levels but different orders). The y-axis represents the value of *P_i _*, which could also be considered as the confidence level for selecting the corresponding *D_i _* value as distance for species discrimination. Strictly speaking, the range of *D_i _* with the corresponding *Pi* ≤ 50% (the performance of random choice) should be filtered out. In order to avoid the unstable part of the curves with undulation, we filter out the range from 0% to 60% to achieve more solid and better performance, even though we consider the validity for the datasets with closer taxonomic similarity.

As a result, for removing the two major kinds of *l*-mer noises (the interspecies and intraspecies noise), only the *D_i _* values with relative positions in the sorted value list from 60% to 80% are used in calculating distance between DNA fragments. Based on traditional Manhattan distance , we define *Modified Chebychev Distance* by restricting these *D_i _* value, where relative positions range from 60% to 80% (the indexes of *a_i _* and *b_i _* are reassigned according to the increasing order of the *D_i _* values):

N(*l*) is the number of *l*-mers described in Methods Section (subsection of *l*-mer frequency calculation).

### *k*-mean clustering and optimization

A clustering algorithm is then applied to classify the vectors into suitable taxonomical groups based on the modified Chebychev distance. As mentioned before, the *l*-mer frequencies feature vectors of DNA fragments from the same species tend to have similar distributions. The *l*-mers distribution within one species is quite stable. Our observation is that, the *l*-mer feature vectors from the same taxonomic sub tree tend to be located around the same clustering center. We use the *k*-mean algorithm to cluster the fragments.

Suppose that we want to cluster the *l*-mers feature vectors of fragments into *k* groups. Based on our distance definition, the objective function of *k*-mean is:

The vector *u_i _* represents the center of the group *S_i _*. We use the traditional method to do the clustering. The processes are described as below:

1. Compute the *l*-mers feature vector for each fragment.

2. Randomly select *k* vectors, each as the center of a group.

3. Cluster all the vectors to the nearest center.

4. For each group, calculate the mean of all the vectors to generate a new clustering center.

5. Repeat step 3 and step 4 many times, say *M* times until the clustering groups are stable.

Because of the unstable feature of *k*-mean caused by the random selection of the initial clustering centers, we will run the algorithm several times with different initial clustering centers and choose the best clustering result with minimum objective value.

## Results

### Testing datasets

For binning methods based on the DNA composition features [15] related, there are four major factors which usually affect binning performance: (1) taxonomic complexity (the number of species in the metagenome dataset), (2) length of the input DNA fragments, (3) sequencing error rate and (4) relative abundance ratio (the ratio of DNA fragments among different species in the metagenome dataset). In our experiment, we used simulated datasets with two to eight species, DNA fragments of length from 500bp to 5,000bp, the sequencing error rate from 1% to 5% and the species relative abundance ratio from the simplest 1:1 to 1:8.

The complete reference genomes of 23 species were downloaded from NCBI genomes database ftp server (http://ftp.ncbi.nih.gov/genomes/). The detailed taxonomic level information of these 23 species was obtained from the NCBI taxonomic database (http://www.ncbi.nlm.nih.gov/taxonomy). They are from three major kingdoms (6 from Archaea, 15 from Bacteria and 2 from Eukaryota).

We implemented our method using C++ (See additional file 1 for the source code and additional file 2 for the user manual.) with two sample testing datasets (additional file 3 and additional file 4) for demonstration under Linux OS environment (Ubuntu 8.10 AMD64 and Debian 5.0 AMD64).

### Clustering accuracy

Since our approach is unsupervised, we assume that we do not know any information about the species that exist in the sample. For each dataset, we compute the accuracy as the percentage of fragments from the same species that are classified in the same group. The exact estimated number of species inside the sample is not necessary in our approach. If the selected *k* value is less than the actual number of species inside the sample, the most similar species will be clustered together into some taxonomic specific groups. If the *k* value is larger than the actual number of species inside the sample, then some special functional genome regions (such as promoters and exogenous transferred regions) will be binned into some taxonomic homologous specific groups. We tested different *k* values for some datasets. And the result was robust. In order to give a fair evaluation of our approach, the following experiments and performances are based on the assumption that the number of species is known for each sample.

In the following, we give a general description and some detailed discussion on the performance of our approach by varying the DNA fragment lengths, the relative abundance ratios of the species, and sequencing error rates.

### General clustering performance

Over 550 datasets were generated. We divided these datasets of microorganisms into three major taxonomic ranges: (1) the same Family but different Genuses (2) the same order but different Families (3) the same Class or higher taxonomical levels but different Orders. The average performances of these datasets with different species complexities (from 2 to 8 microorganisms in one sample) are listed in Table 1. where the same ratio for each species inside the sample and all fragments were assumed to be error-free and of equal length of 2000bp. Table 1 shows that our approach could bin the unknown species with high accuracy and resolution (the capacity to distinguish species with taxonomic difference in classification, i.e. Genus or Family). For example, “Genus” means that these two microbes belong to the same Family but different Genuses; “Family” means that they belong to the same Order but different Families. For each taxonomical group, we show its average, maximum and minimum accuracy among the test datasets. For the taxonomical group of “Genus”, the binning accuracy is higher than 90%. For higher taxonomical differentia, the accuracy increases. When the resolution is Order or higher, the binning accuracy is higher than 99%, this accuracy is comparable with most widely used supervised or semi-supervised binning tools [21,24,25]. The number of microbes inside the samples could also affects the binning performance. When the number of microbes increases, the binning accuracy decreases as the complexity of the sample increases.

**Table 1:** General performance based on different sample complexity and taxonomic similarities

Taxonomic Difference of Species	No. of species in datasets	Accuracy Ave	Accuracy Min	Accuracy Max
**Genus**	2	94.19%	92.88%	95.83%
	3	86.87%	79.83%	90.37%
**Family**	2	98.02%	96.42%	99.57%
	3	96.34%	93.45%	97.90%
**Higher than Order**	2	98.34%	96.45%	99.97%
	3	97.07%	93.22%	99.82%
	4	96.25%	95.56%	98.46%
	6	92.05%	84.68%	96.01%
	8	88.70%	74.57%	96.29%

All the datasets in this table are generated with DNA fragments length equal to 2000bp and 0% sequencing error. The amount of each microbial component in these datasets is equal.

### Different DNA fragment lengths

**Figure 4:**
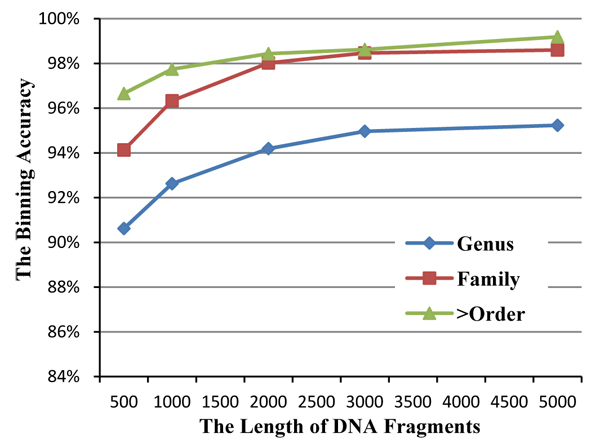
**The binning accuracy based on different length of DNA fragments** For each group of selected genomes, 5 datasets are generated with different fragment lengths (500bp, 1000bp, 2000bp, 3000bp and 5000bp). With the increasing DNA fragment length, the average clustering accuracies based on three major taxonomic ranks tend to increase until a reasonable length is attained.

For metagenomic binning methods based on the DNA composition feature (i.e. feature vector), the DNA fragment length also affects the performance significantly. According to previous research, the performance of binning is improved with longer DNA fragments [24,25] because longer DNA fragments provide more *l*-mers which provide statistically more stable *l*-mer occurrence frequencies. Take 4-mer as an example, the 4-mer frequency vector has 136 components. With DNA fragments of length 500bp, total number of 4-mers is 497 and the average frequency for each vector component is less than 4. Compared to another DNA fragment of length 5000bp, total number of 4-mers is 4997, and the average frequency for each vector component is ten times larger than the previous case. With sufficiently large *l*-mer frequency, the variation of the *l*-mer frequency distribution can be significantly decreased, and the binning accuracy be increased. As shown in Figure 4, there is an obvious improvement in accuracy for our experiment from 500bp to 1000bp. However, once the fragment is long enough, the accuracy improvement will taper off with the increase of fragment length. The recommended length of the DNA fragment is related to the length of *l*-mers i.e. value of *l*. In our case, 2000bp seems to be a reasonable choice.

### Relative abundance ratio of species

**Figure 5:**
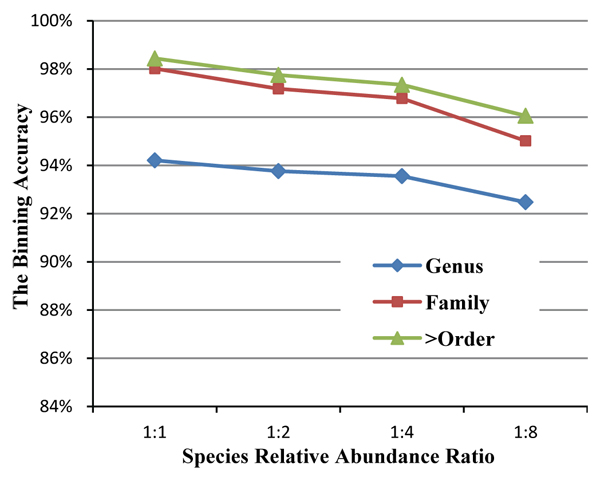
**The binning accuracy based on different species relative abundance ratio** With the increasing of the relative abundance ratio of each group, the average clustering performances based on three major taxonomic ranges tend to decrease.

Species relative abundance ratio can be considered as another factor that affects sample complexity. It is expected that accuracy will decrease if we increase the relative abundance ratio of the species inside the sample. The performance based on the three taxonomical differentials will decrease if we increase the abundance ratio (Figure 5).

**Figure 6:**
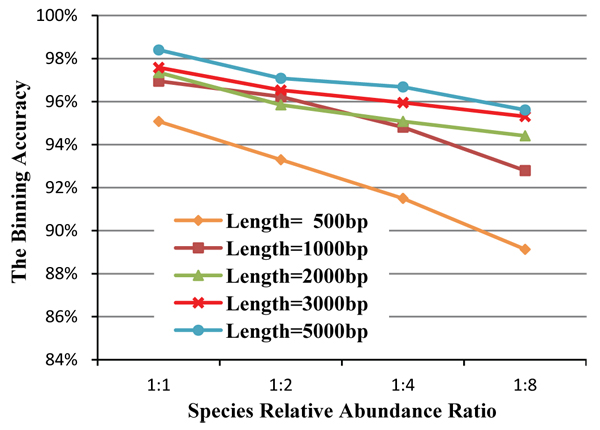
**The binning accuracy based on different species relative abundance ratio and length of DNA fragments.** Within the same dataset, the clustering performance based on longer input DNA fragments is more stable when the relative abundance ratio increases.

We also vary the length of the DNA fragment to analyze whether it helps if the relative abundance ratio is increased in the species. The genomes of two species (*Archaeoglobus fulgidus* and* Methanoculleus marisnigri*), which belong to the same Phylum but different Class, are selected to generate 4 groups of datasets with the relative species abundance ratios 1:1, 1:2, 1:4 and 1:8. Each group contains 5 datasets with different DNA fragments lengths, namely 500bp, 1000bp, 2000bp, 3000bp and 5000bp. Twenty datasets were tested and the 5 curves in Figure 6 represent the performance for the 5 different lengths of DNA fragments and the corresponding 4 abundance ratios. With the increasing of abundance ratio complexity, the binning accuracy is decreased by about 2% to 8% depending on different fragment lengths. Although increasing the fragment length cannot help in maintaining the same accuracy when the ratio increases, we still found that the datasets with longer fragments are more robust for higher abundance ratios. The performance of the dataset with fragment length 500bp decreases by more than 8% when the abundance ratio changes from 1:1 to 1:8. Accordingly, the performance decreases by about 5% for the dataset with fragment length 1000bp, and 2 to 3% for the dataset with fragment length 2000bp.

This result also indicates that the accuracy improvement by increasing the fragment length will taper off from 2000bp to 5000bp.

### Robustness with respect to sequencing error

**Figure 7:**
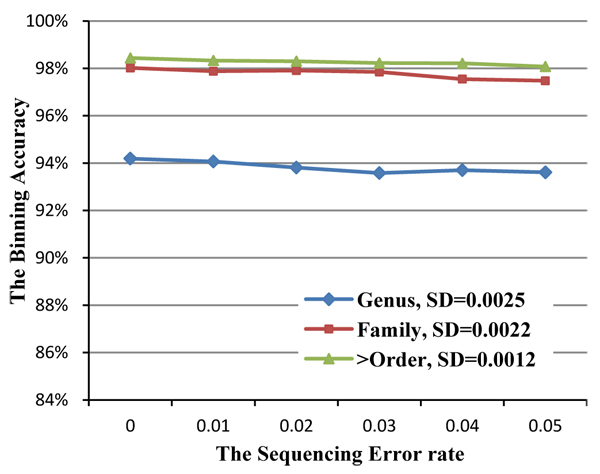
**The robustness of binning accuracy.** The three curves in the figure describe the binning average performance when the sequencing error increases from 0% to 5%. The error bars demonstrate the stability of the binning accuracy.

Sequencing error is inevitable for metagenomics sequencing projects. Hence, error robustness is another important contribution for a successful binning approach. Thus sequencing error is introduced in the simulated metagenomics sequencing datasets. Although the typical sequencing error rate of the existing commercial sequencing platforms is less than 2%, we generated test datasets with error rates ranging from 0% to 5%. The average binning performance for different datasets is illustrated in Figure 7.

The result shows that our method is robust to sequencing errors. Even for the datasets with 5% error, compared with the error free datasets, the accuracy decreases by less than 1%. The error robustness property could be due to the chosen DNA composition features. Take 4-mer as an example: if there is one nucleotide sequencing error, only 4 of the total 4-mers will be affected by this nucleotide sequencing error. So if the sequencing error rate is 1%, for DNA fragments of length 2000bp, the worst case is when the erroneous nucleotides are apart from each other with at least 6 correct nucleotides; hence, no more than 80 erroneous 4-mers are introduced into the 4-mer occurrence frequency calculation. The modified Chebychev distance with sorting and range-picking strategy could effectively remove the effect of these erroneous 4-mers.

## Conclusion and discussion

In this paper, we have introduced an unsupervised DNA composition feature based metagenomic sequencing data binning algorithm focused on the high accuracy taxonomic clustering of unknown species without any reference or markers. Our approach can filter out the interspecies and intraspecies noise to achieve better binning performance and the results are robust even when there are 5% sequencing errors in the DNA fragments. We will improve our method as follows.

### The *l*-value selection of the *l*-mer

The DNA composition features [15] of the occurrence frequency of short oligonucleotides have been reported in previous research from 2-mers to 8-mers. The selection of suitable substring length *l* depends on many factors and it is not sure that large *l* will give better results. When *l* is large, say *l* = 8, there are many (32,768) *l*-mers and the accumulated background noise is so large that we cannot cluster the DNA fragments well. When *l* is small, say *l* = 2, the signal in effective *l*-mers, *l* > 2, will be mixed with the noise in background *l*-mers (*l*-mers with similar frequency in different genomes) so that we cannot cluster the DNA fragments well. For practice, the performance of our approach performs well when *l* = 4 to 6 for most datasets. In the future, we will study how to select a suitable *l* for different datasets according to the error rate, DNA fragment length and the expected similarity of the genomes.

### Clustering algorithm

The traditional *k*-mean clustering algorithm is selected based on the assumption that the distribution of the *l*-mer feature vectors in the hyper-dimensional space is a sphere. When the abundance ratio of different species is extremely different, density-based clustering methods may perform better. Besides, tree structure taxonomic classification among these groups is also important for metagenomic research. Thus, hierarchal clustering methods should be more suitable. In the future, we will study the performance of different clustering algorithms on the binning problem.

### Clustering functional fragments

Another important direction of metagenomic research is that we do not try to identify different species in the sample. Instead we can treat the sample as a single species with very complex genome structure and study which functions can be provided by this genome structure. The *l*-mer frequency may also be suitable for clustering DNA fragments according to their functions.

## Competing interests

The authors declare that they have no competing interests.

## Authors' contributions

B.Y. developed the algorithm and performed the testing and experiments. Y.P., H.L. and S.Y. consummated the testing cases and helped to improve the performance. J.C. implemented the online availability of this tool. F.C. conceived this study. All authors contributed to writing the article.

## Supplementary Material

Additional file 1 This rar package contains the C++ source code of our software. Decompress the package under Ubuntu or Debian environment, then run “Makefile” to install the software. Click here for file

Additional file 2 The pdf file introduces the runtime environment, input & output and the command format of our software. Click here for file

Additional file 3 This rar package contains 4000 DNA fragments from two microorganisms which belong to the same class but different orders. The first 2000 DNA fragments of this dataset are from "Ignicoccus hospitalis KIN4/I" and the last 2000 DNA fragments from "Caldivirga maquilingensis IC-167". The length of each DNA fragment is 2000bp and the sequencing error rate is 2%. Click here for file

Additional file 4 This rar package contains 6000 DNA fragments from three microorganisms which belongs to the same phylum but different orders and classes. The first 2000 DNA fragments of this dataset are from "Bordetella avium 197N", the middle 2000 DNA fragments from "Bordetella parapertussis 12822" and the last 2000 DNA fragments from "Escherichia coli O157:H7 str. Sakai". The length of each DNA fragment is 2000bp and the sequencing error rate is 2%. Click here for file

## References

[B1] Cobb CM Microbes, inflammation, scaling and root planing, and the periodontal condition J Dent Hyg 2008 82 Suppl 3 4 9 19275822

[B2] Khachatryan ZA Ktsoyan ZA Manukyan GP Kelly D Ghazaryan KA Aminov RI Predominant role of host genetics in controlling the composition of gut microbiota PLoS One 2008 3 8 e3064 18725973 10.1371/journal.pone.0003064 PMC2516932

[B3] Amann RI Binder BJ Olson RJ Chisholm SW Devereux R Stahl DA Combination of 16S rRNA-targeted oligonucleotide probes with flow cytometry for analyzing mixed microbial populations Appl Environ Microbiol 1990 56 6 1919 1925 2200342 10.1128/aem.56.6.1919-1925.1990 PMC184531

[B4] Venter JC Remington K Heidelberg JF Halpern AL Rusch D Eisen JA Wu D Paulsen I Nelson KE Nelson W Environmental genome shotgun sequencing of the Sargasso Sea Science 2004 304 5667 66 74 15001713 10.1126/science.1093857

[B5] Tyson GW Chapman J Hugenholtz P Allen EE Ram RJ Richardson PM Solovyev VV Rubin EM Rokhsar DS Banfield JF Community structure and metabolism through reconstruction of microbial genomes from the environment Nature 2004 428 6978 37 43 14961025 10.1038/nature02340

[B6] Jones BV Begley M Hill C Gahan CG Marchesi JR Functional and comparative metagenomic analysis of bile salt hydrolase activity in the human gut microbiome Proc Natl Acad Sci U S A 2008 105 36 13580 13585 18757757 10.1073/pnas.0804437105 PMC2533232

[B7] Mavromatis K Ivanova N Barry K Shapiro H Goltsman E McHardy AC Rigoutsos I Salamov A Korzeniewski F Land M Use of simulated data sets to evaluate the fidelity of metagenomic processing methods Nat Methods 2007 4 6 495 500 17468765 10.1038/nmeth1043

[B8] Altschul SF Madden TL Schaffer AA Zhang J Zhang Z Miller W Lipman DJ Gapped BLAST and PSI-BLAST: a new generation of protein database search programs Nucleic Acids Res 1997 25 17 3389 3402 9254694 10.1093/nar/25.17.3389 PMC146917

[B9] Huson DH Auch AF Qi J Schuster SC MEGAN analysis of metagenomic data Genome Res 2007 17 3 377 386 17255551 10.1101/gr.5969107 PMC1800929

[B10] Cole JR Chai B Farris RJ Wang Q Kulam SA McGarrell DM Garrity GM Tiedje JM The Ribosomal Database Project (RDP-II): sequences and tools for high-throughput rRNA analysis Nucleic Acids Res 2005 33 Database issue D294 296 15608200 10.1093/nar/gki038 PMC539992

[B11] Garcia Martin H Ivanova N Kunin V Warnecke F Barry KW McHardy AC Yeates C He S Salamov AA Szeto E Metagenomic analysis of two enhanced biological phosphorus removal (EBPR) sludge communities Nat Biotechnol 2006 24 10 1263 1269 16998472 10.1038/nbt1247

[B12] Tringe SG von Mering C Kobayashi A Salamov AA Chen K Chang HW Podar M Short JM Mathur EJ Detter JC Comparative metagenomics of microbial communities Science 2005 308 5721 554 557 15845853 10.1126/science.1107851

[B13] Case RJ Boucher Y Dahllof I Holmstrom C Doolittle WF Kjelleberg S Use of 16S rRNA and rpoB genes as molecular markers for microbial ecology studies Appl Environ Microbiol 2007 73 1 278 288 17071787 10.1128/AEM.01177-06 PMC1797146

[B14] Desnues C Rodriguez-Brito B Rayhawk S Kelley S Tran T Haynes M Liu H Furlan M Wegley L Chau B Biodiversity and biogeography of phages in modern stromatolites and thrombolites Nature 2008 452 7185 340 343 18311127 10.1038/nature06735

[B15] Karlin S Burge C Dinucleotide relative abundance extremes: a genomic signature Trends Genet 1995 11 7 283 290 7482779 10.1016/s0168-9525(00)89076-9

[B16] Karlin S Burge C Campbell AM Statistical analyses of counts and distributions of restriction sites in DNA sequences Nucleic Acids Res 1992 20 6 1363 1370 1313968 10.1093/nar/20.6.1363 PMC312184

[B17] Karlin S Ladunga I Comparisons of eukaryotic genomic sequences Proc Natl Acad Sci U S A 1994 91 26 12832 12836 7809130 10.1073/pnas.91.26.12832 PMC45534

[B18] Rubin GM Yandell MD Wortman JR Gabor Miklos GL Nelson CR Hariharan IK Fortini ME Li PW Apweiler R Fleischmann W Comparative genomics of the eukaryotes Science 2000 287 5461 2204 2215 10731134 10.1126/science.287.5461.2204 PMC2754258

[B19] Sandberg R Winberg G Branden CI Kaske A Ernberg I Coster J Capturing whole-genome characteristics in short sequences using a naive Bayesian classifier Genome Res 2001 11 8 1404 1409 11483581 10.1101/gr.186401 PMC311094

[B20] Karlin S Mrazek J Campbell AM Compositional biases of bacterial genomes and evolutionary implications J Bacteriol 1997 179 12 3899 3913 9190805 10.1128/jb.179.12.3899-3913.1997 PMC179198

[B21] Chatterji S Yamazaki I Bai ZJ Eisen JA CompostBin: A DNA composition-based algorithm for binning environmental shotgun reads Research in Computational Molecular Biology, Proceedings 2008 4955 17 28

[B22] Teeling H Meyerdierks A Bauer M Amann R Glockner FO Application of tetranucleotide frequencies for the assignment of genomic fragments Environ Microbiol 2004 6 9 938 947 15305919 10.1111/j.1462-2920.2004.00624.x

[B23] Teeling H Waldmann J Lombardot T Bauer M Glockner FO TETRA: a web-service and a stand-alone program for the analysis and comparison of tetranucleotide usage patterns in DNA sequences BMC Bioinformatics 2004 5 163 15507136 10.1186/1471-2105-5-163 PMC529438

[B24] McHardy AC Martin HG Tsirigos A Hugenholtz P Rigoutsos I Accurate phylogenetic classification of variable-length DNA fragments Nat Methods 2007 4 1 63 72 17179938 10.1038/nmeth976

[B25] Diaz NN Krause L Goesmann A Niehaus K Nattkemper TW TACOA: taxonomic classification of environmental genomic fragments using a kernelized nearest neighbor approach BMC Bioinformatics 2009 10 56 19210774 10.1186/1471-2105-10-56 PMC2653487

