# Supplementary material for: Unsupervised binning of environmental genomic fragments based on an error robust selection of l-mers
Source: BMC Bioinformatics. 2010 Apr 16;11(Suppl 2):S5. doi: 10.1186/1471-2105-11-S2-S5 (PMC3165929; doi:10.1186/1471-2105-11-S2-S5)
Supplement: Additional file 2 — The pdf file introduces the runtime environment, input & output and the command format of our software. [file 1471-2105-11-S2-S5-S2.pdf]

Version 0.1 ***beta***

MetaCluster

User manual v0.1 *beta*

Released on Jan 21st 2010

Outline

1 File Information
2 Runtime Environment
3 Input & Output
4 Command Format

For research and academic use

All copyrights for commercial application reserved

File Information

The version 0.1 *beta* release package contains the C++ source code of "`MetaCluster_0.1_beta`" and one PDF version user manual: "`MetaCluster_Manual.pdf`". You can also check our website to download simple examples: http://www.cs.hku.hk/~alse/MetaCluster

Runtime Environment

64-bit processor and 4G or more memory is very necessary for better performance or solving large dataset (over 10,000 DNA fragments and 100,000 is the maximum value). MetaCluster is developed and tested under Ubuntu 8.10 AMD 64 version and Debian 5.0 AMD 64 version and should be suitable for all Linux environment.

Input & Output

The input of MetaCluster should be a sequencing dataset contain several DNA fragments with FASTA format like:

```
>read0001
TACGCGGACTTCGATGCG … GCTAGGCTGACG
>read0002
GCCTGCGAAGCGCGATTT … AGCTGAGGCGTC
```

An estimated approximate cluster number say *n*, is another input. And according to this value, the MetaCluster will divide the input dataset to *n* taxonomic specific output files with FASTA format as the output under the same path of the binary file, like:

```
gourp0.fasta; group1.fasta …
```

Command Format

The typical command format of MetaCluster is:

```
$ MetaCluster input_file [--classes n]
```

The default value of *n* is 2.

Copyright© 2009-2010, Department of Computer Science | The University of Hong Kong
